# IMPACT OF AN ONLINE EMOTION-FOCUSED COMMUNICATION TRAINING ON STAFF KNOWLEDGE AND SELF-EFFICACY

**DOI:** 10.1093/geroni/igac059.1848

**Published:** 2022-12-20

**Authors:** Katherine Abbott, Allison Heid, Megan Kelley, Alexandra Heppner, Kimberly Van Haitsma

**Affiliations:** Miami University, Oxford, Ohio, United States; Ardmore, Pennsylvania, United States; Miami University, Oxford, Ohio, United States; Miami University, Oxford, Ohio, United States; Pennsylvania State University, University Park, Pennsylvania, United States

## Abstract

The way we respond emotionally to others can impact how we provide care. Emotional intelligence is vital for care team members whose entire day involves interacting with other people. We have developed an interactive, online, self-paced course for people providing care to others with the specific goal of increasing awareness of emotions and helping to identify emotions in others. The training is supported by research indicating that individuals who can better manage their own emotions are better positioned to manage behaviors and emotions in others. The central concept of the training is to build relationships that enhance person-centered care through increasing care providers’ emotional intelligence. These skills allow care providers to better manage their own behavior and emotions, which results in improved quality of their care work. The purpose of this study was to assess the knowledge, self-efficacy, acceptability, and appropriateness of the online training. The data for this study came from n = 130 individuals (19% direct care worker, 16% activities, 13% health care provider, and 12% case manager) who completed assessments pre-and post-training. Knowledge of emotion-focused communication strategies and self-efficacy in using emotion-focused communication strategies both increased significantly with training (t(129) = -5.40 p < .001 and t(124) = -6.42 p < .001). In addition, high levels of acceptability, appropriateness, and satisfaction were reported. Findings indicate the benefits of online training for emotion-focused communication for caregivers. The discussion will focus on recommendations for practice, policy, and research.

